# Assessing the Impact of Spatial and Temporal Variability in Fine Particulate Matter Pollution on Respiratory Health Outcomes in Asthma and COPD Patients

**DOI:** 10.3390/jpm14080833

**Published:** 2024-08-06

**Authors:** Irini Xydi, Georgios Saharidis, Georgios Kalantzis, Ioannis Pantazopoulos, Konstantinos I. Gourgoulianis, Ourania S. Kotsiou

**Affiliations:** 1 Department of Respiratory Medicine, Faculty of Medicine, University of Thessaly, 41110 Larissa, Greece; eirinixydi@uth.gr (I.X.); kgourg@med.uth.gr (K.I.G.); 2 Department of Mechanical Engineering, University of Thessaly, 38334 Volos, Greece; saharidis@uth.gr (G.S.); george.kalantzis4@gmail.com (G.K.); 3 Emergency Medicine Department, Faculty of Medicine, University of Thessaly, 41110 Larissa, Greece; ipantazop@uth.gr; 4 Laboratory of Human Pathophysiology, Nursing Department, University of Thessaly, 41500 Larissa, Greece

**Keywords:** fine particulate matter (PM_2.5_), indoor air quality, indoor-to-outdoor (I/O) ratio, spatio-temporal variation, respiratory health, asthma, COPD

## Abstract

Ambient air pollution’s health impacts are well documented, yet the domestic environment remains underexplored. We aimed to compare indoor versus outdoor (I/O) air quality and estimate the association between indoor/ambient fine particulate matter (PM_2.5_) exposure and lung function in asthma and chronic obstructive pulmonary disease (COPD) patients. The study involved 24 h monitoring of PM_2.5_ levels indoors and outdoors, daily peak expiratory flow (PEF), and biweekly symptoms collection from five patients with asthma and COPD (average age of 50 years, 40% male) over a whole year. Data analysis was performed with linear mixed effect models for PEF and generalized estimating equations (GEE) for exacerbations. More than 5 million PM_2.5_ exposure and meteorological data were collected, demonstrating significant I/O PM_2.5_ ratio variability with an average ratio of 2.20 (±2.10). Identified indoor PM_2.5_ sources included tobacco use, open fireplaces, and cooking, resulting in average indoor PM_2.5_ concentrations of 63.89 μg/m^3^ (±68.41), significantly exceeding revised World Health Organization (WHO) guidelines. Analysis indicated a correlation between ambient PM_2.5_ levels and decreased PEF over 0-to-3-day lag, with autumn indoor exposure significantly impacting PEF and wheezing. The study underscores the need to incorporate domestic air quality into public health research and policy-making. A personalized approach is required depending on the living conditions, taking into account the exposure to particulate pollution.

## 1. Introduction

An increasing number of the world’s population is gathering in urban centers at faster than ever rates, and it is projected that by 2050, 68% of people will live in cities [1]. At the same time, data suggest a more substantial prevalence of obstructive lung diseases such as asthma and chronic obstructive pulmonary disease (COPD) in urban populations [2]. The WHO reports that poor air quality, which is a major driver of chronic respiratory disease in urban areas [3], was related to 7 million premature deaths worldwide [4]. The association between exposure to ambient air pollution and mortality, hospital admissions and lung function is well documented [5,6,7]. Among air pollutants, particulate matter, PM, which is a mixture of solid particles and liquid droplets found in the air [7], is linked to respiratory and cardiovascular diseases, as well as to mortality and morbidity [8,9,10]. Fine particles, known as PM_2.5_ (≤2.5 μm in aerodynamic diameter), are now used as a main indicator of risk in the air quality indices (AQIs) in many countries [11]. Ambient PM_2.5_ is mainly generated through combustion of gasoline, oil, diesel fuel, or wood. At the same time, air quality inside the home environment may be affected by anthropogenic PM_2.5_ emissions related to residents’ behavioral patterns, i.e., type of cooking and heating, ventilation, environmental tobacco smoke (ETS), etc., whereas infiltration from outdoor sources plays a contributing role. Because of their small diameter, PM_2.5_ is able to infiltrate through the nose into the lower respiratory system, accumulating in respiratory bronchioles [12]. Alveolar damage may consequently occur due to the production of free radicals, imbalanced intracellular homeostasis, and inflammation [12].

The effect of outdoor particle pollution on lung function and symptoms has been extensively investigated in numerous panel studies [13,14,15,16]. Few epidemiological studies have focused on the health effect of indoor air quality on patients with chronic respiratory diseases, such as asthma and COPD. Studies concerning the indoor environment focus on a number of diverse micro-environments; however, a review highlights that epidemiological results focusing on dwellings of asthmatic subjects are less conclusive and acknowledges that further research is needed to clarify the strength of the relationship between indoor exposure to particles and asthma [17]. Home environment, where people spend the majority of their time [18,19], is of particular importance when assessing the health effect of air pollution. Taking this under consideration, there is an increasing interest in global research for indoor air quality (IAQ); however, existing results on air pollution in the home environment and lung function are inconclusive. At the same time, there is a limited availability of objective measurements of indoor PM_2.5_ to accurately assess their effects on respiratory health. Furthermore, indoor air quality monitoring is carried out for a relatively short period of time, ranging from a few days to a few months for each residence, thus potentially misrepresenting the real level of exposure.

Hart et al. found that low-level exposures to indoor PM_2.5_ of 125 COPD patients from Eastern Massachusetts, USA, that were monitored for one week each season over a year were associated with decreases in lung function [20]. A study, carried out in four European cities (Helsinki, Athens, Amsterdam, and Birmingham), has shown no association between indoor and outdoor particulate matter concentrations and the pulmonary function of asthma and COPD patients [21]. In that study, concentration and health outcome data were collected three times a day for 1 week through an over-a-year study period. In a study performed in two cities in the USA, no significant associations were observed with increasing concentrations of PM_2.5_ [22]. The study included 24 asthma and/or COPD patients, nine of whom resided in New York City and 15 in Seattle, and measurements were carried out for 12 days during winter and summer at the participants residences. In another study conducted in the city of Mexico with a panel of 29 COPD patients, a significant increase in cough and phlegm was found for a 10 μg/m^3^ increase in personal exposure to PM_2.5_, with a 2-day lag, together with a significant reduction in the morning and evening peak expiratory flow (PEF) for the same time lag [23]. For the exposure measurement, study subjects carried personal monitors for 12 consecutive days, and the follow-up period was repeated three times through the year. Evangelopoulos et al. found no association between particulate matter and respiratory health outcomes in a study carried out in London among COPD patients [24]. Personal exposure to various air pollutants, with PM_2.5_ being one of them, was monitored for an average period of 128 days. Paterson reviewed 12 epidemiological studies based on objective measurements of PM_2.5_ and found insufficient evidence to determine the effect of fine particles on asthmatic adults in the home environment [25].

While ambient air pollution has been investigated for many years, there are still gaps in our understanding of the magnitude of peak exposure to air pollutants, especially the most hazardous ones, such as PM_2.5_, in the domestic micro-environment. This research aims to provide a quantitative contribution to the existing findings on the relationship between respiratory status and fine-particle exposure in a mid-sized city of Southeastern Europe, a geographical area that has received minimal research attention. The present work was designed to test the hypothesis that personal PM_2.5_ exposure, especially in the domestic environment, contributes to aggravating respiratory health in vulnerable population groups, such as asthmatic and COPD patients. By collecting an extended amount of respiratory health indicators per patient, such as PEF and symptoms, this research provides an insight on the extent to which PM_2.5_ exposure is linked to lung function while patients are still at home, in contrast to most other published work, which focuses on hospital admissions or emergency department visits. This time-series prospective study had two distinct objectives. First, to evaluate the relationship between outdoor and indoor concentrations of PM_2.5_ and short-term effects on the respiratory health of adult asthmatics and COPD patients. Second, to delineate the relationship between real-time, objective measures of domestic, indoor, and immediate-outdoor PM_2.5_ concentration levels and their temporal variability over a monitoring period of an entire year. Understanding these relationships can lead to improved public health strategies, more effective air quality guidelines, and targeted interventions aimed at reducing indoor pollution. Consequently, this could result in better management and prevention of chronic respiratory diseases, enhancing the quality of life for affected individuals.

## 2. Materials and Methods

### 2.1. Study Design

A time-series panel study was conducted in the medium-sized city of Larissa, Region of Thessaly, Greece. Larissa [~149.600 population (2021)], located on the mainland of central Greece, is extended on a mild ground terrain, surrounded by mountainous relief, and is characterized by a continental climate. One of the main objectives of the study was to capture seasonal variations of PM_2.5_ concentrations, both in the ambient and indoor environments of the residences. To this end, the study period extended over a whole calendar year, from 15 November 2021 to 14 November 2022. Residences in the vicinity of different PM emission sources (i.e., main traffic junctions, central transportation stations, suburban areas, etc.) were identified and selected. For the objective measurement of PM_2.5_ exposure, monitoring devices were installed in the indoor and immediate outdoor environments of the selected residences. Lung-function was assessed with peak expiratory flow (PEF) measurements and symptoms of exacerbations. The association between PEF changes per unit increase in 24 h mean PM_2.5_ concentrations was estimated by employing linear mixed-effect models, while symptoms exacerbations were estimated by using generalized estimating equations (GEEs). The study protocol was approved by the Larissa University Hospital Scientific Committee (10/06-07-2021), and all participants signed a written informed consent before the initiation of the study.

### 2.2. Participants Recruitment and Residence Characteristics

Study subjects were enrolled among outpatients that had routinely visited the asthma and COPD clinics of the University General Hospital in Larissa during 2020. The list of contacts was retrieved through an initial screening of the “ASKLIPIOS” hospital database, in which patients with registered contact details and residencies within city limits were included as potential participants. The study focused on asthma or COPD patients as a sub-population that is vulnerable to the effects of particulate air pollutants.

Specific features of the city were taken into consideration, such as the density of urban structure, type of urban zones, locations of bus and train main stations, major traffic junctions, ring roads, and geomorphological characteristics (i.e., proximity to the river, etc.). These areas of interest were identified and geocoded on the GIS program (QGIS, version 3.16), together with patients’ addresses. The number of sensors per area, the distance between the sensors of different areas, and the specific characteristics of each area were taken into consideration. Residences in the proximity of the areas of interest were selected.

A phone communication with candidate subjects followed the aforementioned selection process in order to provide them an overview of the study’s purpose and requirements, as well as to explore their willingness to participate. At the same time, the availability of additional infrastructure requirements, regarding Wi-Fi connection and external power socket, was confirmed.

**Figure 1:**
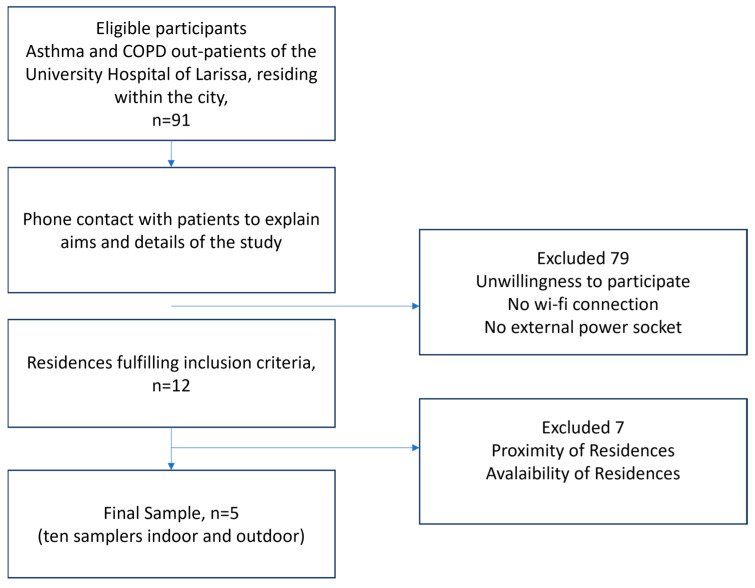
Flowchart of sample selection process.

The flowchart describing the participant-selection process is presented in Figure 1. Due to the intensive follow-up program, which required active participation of the patients for a whole year combined with regular, time-consuming visits by the technical team for installing and maintaining the samplers, positive response to the study was limited. Apart from daily self-measuring and recording of PEF results and answering extensive clinical asthma or COPD questionnaires biweekly, the fact that the sampling period coincided with the outburst of the COVID-19 pandemic heightened the patients’ skepticism. In view of the study requirements, COVID-19 restrictions, and the necessity to protect vulnerable populations, the final sample size of five asthma or COPD patients was defined. It is remarkable that there were no dropouts during the study period.

### 2.3. Respiratory Health

Peak expiratory flow (PEF) was monitored since it is considered a reliable indicator of ventilation adequacy as well as of airflow obstruction. Study subjects took daily PEF measurements with mini-Wright peak flowmeters after extensive instructions regarding their usage were given to them at recruitment. The use of a peak flowmeter was performed every morning in a standing position before the intake of their respiratory medication. Flowmeter measurements were self-recorded by the participants in a personal diary.

The Asthma Control Test (ACT) and Asthma Quality of Life Questionnaire (AQLQ) for asthma patients and the Clinical COPD Questionnaire (CCQ) and St. George’s Respiratory Questionnaire for COPD Patients (SGRQ-C) were completed on a biweekly basis. Asthma and COPD exacerbations were also recorded. Asthma exacerbations are defined as the episodes of progressive increase in shortness of breath, cough, wheezing, or chest tightness, or some combination of these symptoms, accompanied by decreases in expiratory airflow that can be quantified by measurement of lung function [Gina 2010, Gina 2023]. The exacerbation of COPD is defined as an event characterized by dyspnea and/or cough and sputum that worsens in <14 days [GOLD 2023].

### 2.4. PM_2.5_ and Meteorological Factors Monitoring

The monitoring network consisted of ten measuring devices (GreenYourAir Device 1178/PM_2.5_) that were installed both indoors and outdoors. The light-scattering method was utilized for the samplers, as previously described [12]. The main parts of the device are a sensor that provides data for the concertation of PM_2.5_, temperature, and relative humidity, an I/O expansion shield, and an Arduino YUN rev. 2. The programming language of the device is C++. The devices collected data with a 3 min time stamp and were operating 24 h per day.

The GreenYourAir team developed a two-phase calibration methodology to validate the accuracy of the devices. During the first stage of development and testing, the sensors were validated in laboratory conditions using reference equipment that followed EU standards EN 14907:2005 (gravimetric device with filters that collects PM_2.5_) [26]. Later, during the second stage, the sensors were validated in real-life conditions.

Measurements of the concentration of PM_2.5_ (μg/m^3^) and meteorological parameters of temperature (T, °C) and relative humidity (RH, %) in both the indoor and immediate outdoor environments of each residence were recorded, in real time, every 3 min, and transmitted wirelessly to a main server. This way, over 5 million data points were collected during the sampling period and were subsequently checked and aggregated to 24 h means.

### 2.5. Statistical Analysis

The relationship between the subjects’ PEF and indoor and outdoor PM_2.5_ exposure was estimated using linear mixed effects models (LMEMs) with random intercepts. In order to assess the impact of particle pollution exposure, changes in PEF per unit increase in PM_2.5_ concentrations were estimated for the same day (lag 0) as well as for up to 3 previous days (lag 1, lag 2, and lag 3). Temperature is considered a major confounding factor in air pollution epidemiological studies. In this context, the model was adjusted for temperature, together with confounders of gender and season indicators, adding one potential covariate at a time. A first-order autoregressive (AR-1) covariance matrix was selected in order to adjust for auto-correlation between repeated measures on the same subject. In all models, the subject in sampling session was referred to as a random effect. The relationship between the binary categorical variable of symptoms exacerbation (cough, dyspnea, and wheezing) (yes = 1; no = 0) and increasing PM_2.5_ concentrations was analyzed using generalized estimating equations (GEEs). The beta estimates were expressed as change in the expected PEF or as odds ratios for exacerbation for a unit increase in fine particles. Units of PM_2.5_ concentrations were expressed in mass per volume (μg/m^3^) and PEF measurements in volume per time (L/min). The strength of the association was described in terms of *p*-values, with *p* < 0.05 being considered statistically significant. Statistical analysis was performed using the SPSS statistical package (IBM Corp. Released 2016. IBM SPSS Statistics for Windows, Version 24.0. Armonk, NY, USA; IBM Corp).

## 3. Results

During the first home visit, a questionnaire concerning personal data such as date of birth, height, weight, respiratory health history and use of medication, smoking habits, as well as potential occupational exposure to air pollutants, together with various residence characteristics, was filled out. Each one of the five samplers was securely placed in the living room of each residence, making sure they were at a considerable distance from appliances that could potentially affect PM_2.5_ measurements (air conditioning unit, exhaust hood, fireplace, etc.) and five samplers were installed outdoors, on the terrace of each residence. The specific household characteristics for each patient connected with fine PM exposure are depicted in Table 1. The study group consisted of 5 asthma and COPD patients, 3 women and 2 men, aged between 38 and 62 years old [mean age (SD): 51.6 (9.8)], 3 of whom were currently smokers.

### 3.1. Data Collection—PM_2.5_ Exposure

For the 12-month monitoring period between 15 November 2021 and 15 November 2022, more than 5 million fine particle exposure and meteorological data were collected, screened, and aggregated to 24 h daily means. Concurrently, daily PEF measurements were recorded, along with biweekly symptoms of cough, dyspnea, and wheezing and questionnaire completion. Participants spent on average 58% of their time in the home environment (Table 1), ranging from 74% during winter to 48% during summer.

Overall, a total of 1707 PEF readings were obtained out of 1825 person-days, and an average of 282 sampling days per participant [215 (min)–354 (max) days] were included in the analysis. Evidently, there were missing data both in terms of health outcomes as well as of fine particle exposure measurements. PEF measurements in periods when the participants were ill or away from home, were excluded from the dataset. Also, there were periods of time when data transmission from the samplers was interrupted, either due to internet connection problems or device malfunction. Missing data of fine PM exposure or health outcome parameters were not replaced with estimates.

**Figure 2:**
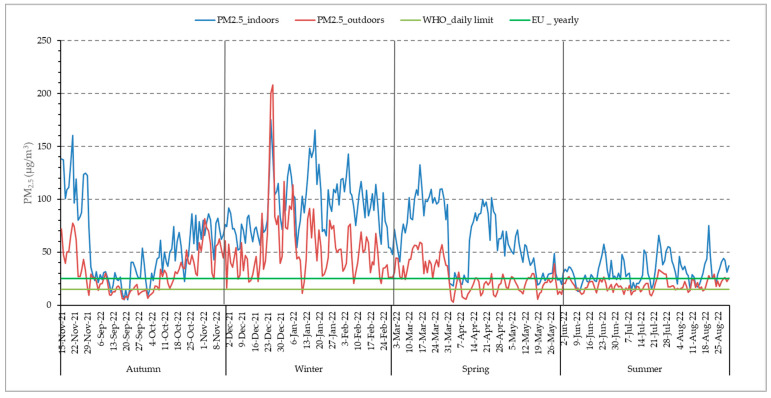
Distribution of daily indoor and outdoor PM_2.5_ concentrations.

The mean annual concentration of ambient PM_2.5_ in the city of Larissa was 33.88 (24.03) μg/m^3^ (revised 2021 yearly WHO guidelines: 5 μg/m^3^) (Table 2). Overall, the revised WHO daily limit of PM_2.5_ (15 μg/m^3^) was exceeded for 298 days and the corresponding EU legislative limit (25 μg/m^3^) was exceeded for 186 days, which was 81.6% and 53.8% of the study period, respectively. The recorded levels of outdoor PM_2.5_ were found to be higher in winter, followed by autumn, spring, and summer (Table 2). Average outdoor PM_2.5_ levels were 7 times above the WHO yearly guidelines, whereas the indoor PM_2.5_ mean annual concentration of 63.89 μg/m^3^ was 13 times above the aforementioned limit. In Figure 2, the temporal variation of PM_2.5_ concentrations is described, both yearly and per season. It is observed that indoor fine particle exposure is higher than the outdoor one and exceeds the WHO guidelines for averagely 248 days of the year. Even during the warm season, despite the increased ventilation of the residences, indoor PM_2.5_ exposure is higher than the outdoor one. It should be noted that in the 2010 indoor air quality report [27,28], WHO states that regarding PM_2.5_ specifically, the same outdoor air quality guidelines apply to the indoor environment as well.

**Figure 3:**
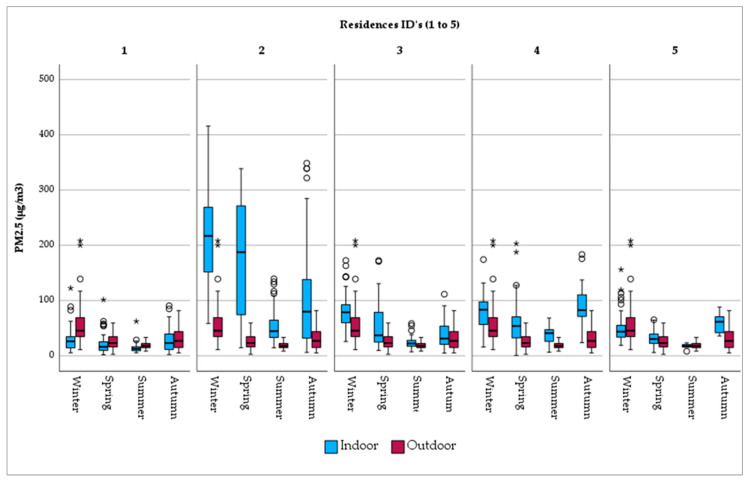
Seasonal variation in PM_2.5_ concentrations by monitoring site. **Note:** There is no difference between the circles. In this boxplot, red circles represent values of outdoor concentrations and blue circles, the respective indoor ones. Now, there are only two categories of outliers. asterisks (*) in this boxplot stand for extreme outliers, i.e., data points that are more extreme than Q3 + 3 * IQR. Circles (○) in this boxplot stand for mild outliers, i.e., data points that are more extreme than Q3 + 1.5 * IQR but are not extreme outliers.

When PM_2.5_ exposure is examined per residence, it is observed that outdoor fine particles are uniformly distributed over the city (Figure 3). A similar pattern does not apply in the home environment, where a considerable range in concentration is found. Indicatively, indoor exposure in Res_2, with the open fireplace together and the presence of tobacco smokers, PM_2.5_ levels are considerably high (mean PM_2.5_ = 137.56 μg/m^3^). Res_1, on the other hand, with limited sources of PM_2.5_ generation (i.e., short cooking duration, no ETS, etc.), is characterized by lower levels of fine particle exposure (mean PM_2.5_ = 22.22 μg/m^3^). The same tendency is observed in the seasonal variation per residence (Figure 3).

**Figure 4:**
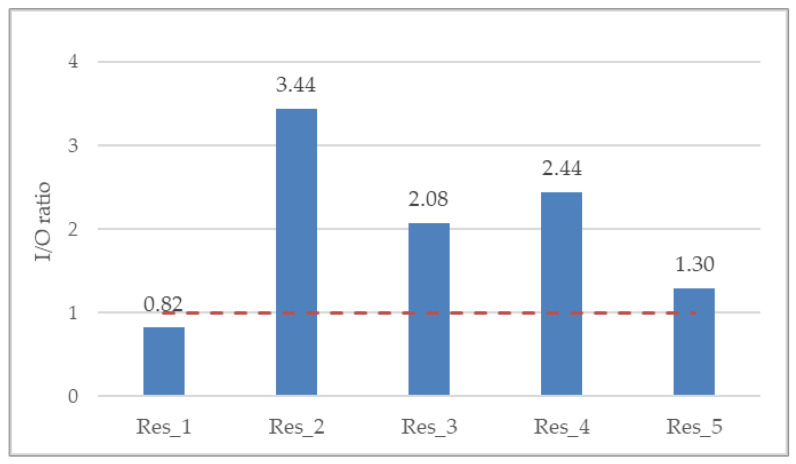
Annual mean indoor-to-outdoor (I/O) ratio for all residences. Red dotted line denotes an I/O ratio of 1, where the indoor PM_2.5_ exposure is equivalent to the outdoor one.

The overall indoor/outdoor (I/O) ratio was 2.13, whereas per residence it ranged from minimum 0.82 (Res_1) to maximum 3.44 (Res_2) (Figure 4). The I/O ratio is suggestive of the ventilation of the indoor space as well as residents’ behavioral patterns. The greater the I/O ratio is than 1 (red dotted line), the more considerable the indoor PM_2.5_ generation anthropogenic activities.

### 3.2. PM_2.5_ Impact on Pulmonary Function

Over the year, a statistically significant decrease in daily PEF recordings was observed, as ambient PM_2.5_ concentration increased for same-day exposure up to three consecutive previous days (Table 3). Despite increased exposure levels in the home environment, a negative correlation was observed only for same-day exposure, but it was not statistically significant. The impact of outdoor PM_2.5_ on PEF decrement may be attributed to specific chemical components of fine particles, which are present due to burning biomass and other low-cost alternatives (melamine surface materials, oil grades, etc.) used for heating purposes. Seasonal ambient PM_2.5_ associations were less consistent (Table 3). A significant negative association between PEF and indoor air quality was found for “lag 0” as well as up to “lag 3” for autumn. Consistently, lung function was worsening during autumn, in the residential environment, with the association being significant for lag 0 up to lag 3. Less consistent associations were not surprising, considering the small number of subjects, as well as the fact that they all suffer from chronic lung diseases, whose day-to-day pulmonary functions exhibit high variability. Furthermore, the absence of significant effects during the other seasons may be due to the lack of adjustment by other air pollutants or effects caused by the typical chemical composition of fine particles, which was not examined in the present study.

PM_2.5_ exposure in the domestic micro-environment was significantly associated with wheezing (Table 4). Applied statistical analysis presented no fine particle impact on biweekly ACT and AQLQ scores (Table 5).

## 4. Discussion

Our research provides an insight into the personal exposure to fine particle pollution and its corresponding effect on reduced peak flow. As mentioned above, PM_2.5_ concentrations were measured on a daily basis over an entire year, both in the indoor and immediate outdoor environments of five selected residences, located in the mid-sized city of Larissa, in central Greece. At the same time, PEF measurements and relevant symptoms of the asthmatic and COPD patients residing in them were recorded. The aforementioned indoor concentration measurements revealed an increased PM_2.5_ exposure, associated with smoking and/or the use of an open fireplace inside the residence. In those cases, a diminished lung function as well as an exacerbation of distinct respiratory symptoms was observed, in accordance with the findings of published research [16,23], although no PM_2.5_ variation impact on biweekly AQLQ and ACT scores was presented.

Participants in the present study spent an overall average of 58% of their time inside their residences, significantly more so during winter (75% on average) than in the summer (48% on average). The observed time spent indoors was relatively low, yet not uncommon for a city in southern Europe, and can be largely attributed to mild weather conditions and the lifestyle of the general population. Relevant studies in developed countries have found that the corresponding average time spent inside the residence was typically around 65% [18,19].

Results of the present study showed a higher outdoor exposure to fine particles in wintertime. This could be mainly attributed to the widespread burning of wood in stoves and fireplaces, which was established as a considerably low-cost way of heating households in Greece during the financial crisis undergone by the country and has remained ever since. The restaurant industry in general, and grill restaurants in particular, which are highly developed throughout Greece and comprise numerous sites of smoke emissions, is considered to be another significant contributor. Their effect in Larissa is generally more pronounced in the winter, since many restaurants in the city suspend their operation in the summer due to the lack of clientele, as a large fraction of the population spends the summer away from it. Finally, vehicle emissions constitute another major source of particulate matter, especially during rush hours, as is usually the case in most urban areas. Apart from anthropogenic activities, elevated PM_2.5_ concentrations during winter are linked to weather conditions and meteorological variables. Meteorological conditions such as atmospheric stagnation [29], temperature inversions, and lower-boundary layer height [30] prevent pollutant dispersion, whereas results from published research reveal a moderate negative correlation between PM_2.5_ and temperature and a positive one between PM_2.5_ and relative humidity [31,32]. As regards spatial variability, no substantial concentration differences among outdoor monitoring sites were found. An even distribution of PM_2.5_ is favored by the level terrain of the study area and is indicative of the absence of localized sources of particle pollution, combined with low deposition rates of fine particles. Unlike the findings of our research, a study conducted in the metropolitan city of Ankara, Türkiye, found considerable variability in ambient PM_2.5_ exposure among eight monitoring sites, mainly due to the city’s varied topography, land-use patterns, and transportation infrastructure [33]. The same study, which collected data on PM_2.5_ concentration levels between the years 2020 and 2022, also found that PM_2.5_ concentrations reached their highest during the winter, with increased residential heating and traffic density being recognized as the main contributing factors.

As regards the domestic environment, the present study found that the 24 h mean PM_2.5_ concentrations were remarkably high in the residence of smokers who also used an open fireplace (Res_2), followed by those with smoking residents but no fireplace, clearly highlighting the role of wood burning and tobacco smoke as major sources of indoor PM_2.5_ emissions. The use of a fireplace resulted in a daily maximum PM_2.5_ indoor level of 459 μg/m^3^, which is very close to the findings of another study, conducted in the U.S.A., where the maximum PM_2.5_ concentration in homes using woodstoves reached 434 μg/m^3^ [34]. In the aforementioned residence of our study, the daily mean PM_2.5_ concentration of 221 μg/m^3^ during the winter practically coincides with the result reported in another research study, which was also conducted in residences of smokers who used biomass as a fuel, where the corresponding PM_2.5_ exposure was 225 μg/m^3^ [35]. In contrast, another study, in which indoor PM_2.5_ exposure was measured in residences employing woodstoves in Oslo, Norway, found that the mean hourly PM_2.5_ concentration did not exceed 16 μg/m^3^ [36].

With respect to seasonal variation, considerable temporal differences were observed. The 24 h average indoor PM_2.5_ concentration during summer was 2 to 3 times lower than in winter in all residences, with the exception of Res_2 (smokers and open fireplace), in which case it was 4 times lower. This finding stresses the beneficial role of increased aeration, as well as the aggravating impact of wood burning on indoor air quality. In winter, PM_2.5_ exposure inside the living room of Res_2 was found to be 7.8 times higher than in Res_1 (non-smokers, brief cooking activities), 2.8 times higher than both Res_3 (smokers) and Res_4 (smokers), and 4.5 times higher than Res_5 (‘‘vapers’’ and long cooking activities). Several studies have indicated an association of the duration of cooking, smoking, and the use of open fireplaces with PM_2.5_ 5 concentrations inside the home. In accordance with the present study, it was found that, inside the bedroom area, PM_2.5_ concentrations were 2.8 times higher in residences with smokers [37]. Nasir et al. [38] observed lower PM_2.5_ concentration levels in smokers’ living rooms in the U.K. during the winter compared to the ones found by the present study. This could be due to the number of cigarettes smoked per day or the time spent outdoors, since the study group of the UK research consisted of university students. In addition, the monitoring period, which only lasted from 1 to 3 weeks, may not have been adequately long in order to reveal the actual level of exposure. Nevertheless, in both studies PM concentrations were higher in smokers’ living rooms than in the case of non-smokers.

A high variability of indoor-to-outdoor (I/O) ratio was observed among the five residences of the present study, ranging from a minimum of 0.8 (Res_1) to a maximum of 3.4 (Res_2), which indicates the effect of indoor PM emission sources. Two other studies in the U.K. reported I/O ratios ranging from 1.0 to 2.3 in five non-smokers’ residences during the warm season [39] and, in agreement with the present study, ranging from 0.8 to 2.8 in both smokers’ and non-smokers’ residences with no fireplace [38]. Furthermore, the I/O ratio observed in the non-smokers’ residence of the present study (Res_1) is in good agreement with the corresponding value reported by Zhao et al. [40], whose research was conducted in Germany and yielded a mean I/O value of 0.76, as derived from relevant measurements in forty non-smokers’ residences. However, unlike the non-smokers’ residence of our study, results from a study carried out in Hong Kong found an average I/O ratio greater than unity, equal to 1.3 in particular, in 27 subjects that participated in concurrent outdoor, indoor, and personal PM_2.5_ monitoring, on two consecutive days in both the summer and winter seasons during 2014–2016 [41].

Increases in the concentration of fine particles in the outdoor environment seem to have significantly affected the lung function of the present study’s subjects. Moreover, significant seasonal associations were found between indoor PM_2.5_ exposure and impaired respiratory health of asthmatic and COPD patients. Results of this study have shown a significant decrease in PEF, for lag 0 up to lag 3, per unit increase in PM_2.5_ concentration during autumn. Finally, PM_2.5_ exposure in the domestic micro-environment was significantly associated with wheezing.

No PM_2.5_ impact on biweekly ACT scores was presented. The lack of an effect on asthma control over time may be related to the complex heterogeneous nature of the disease, whose development is, among others, affected by various environmental exposures such as air pollutants and allergen factors [42,43]. It should also be mentioned that the asthmatic patients of the study received regular maintenance medication and were well-educated regarding self-regulation of their medication dosage as well as their routine activities intensity. Additionally, the estimated lack of an effect on asthma outcomes could also be attributed to the COVID-19 pandemic and the implementation of self-protective measures, such as the use of masks and consistent hand hygiene habits, thus restricting viral respiratory infections, which may, in turn, trigger asthma exacerbation [43].

One of the objectives of the present study has also been to quantify possible changes in the quality of life of asthmatic patients due to personal exposure to fine particulate matter. Applied statistical analysis resulted in no significant difference in biweekly AQLQ scores. Few studies have addressed the question of differences in AQLQ scores in relation to air pollutants concentrations, and their design remotely resembles this one. A recent study examined 50 asthma patients in a placebo-controlled randomized clinical trial, where air purifiers with active filters (intervention) or dummy filters (placebo) were placed in the participants residences [43] Air quality data (PM_10_, PM_2.5_) and health outcomes, among which changes to AQLQ scores, were selected. After 78 weeks, despite improvements in indoor air quality due to air purifiers, the intervention group showed no significant improvement in quality-of-life measures when compared to matched controls. In another study, 300 adult asthma patients filled out the Asthma Quality of Life Questionnaire (AQLQ) once after a 2-week follow-up period, where ambient PM_2.5_ data were obtained from central monitoring stations on a daily basis [44]. Increased PM_2.5_ exposure resulted in significant AQLQ score impairment in all partial domains tested, and, overall, a decrease in the total AQLQ score by 0.16 was assessed.

A study performed in New York City and Seattle, U.S.A., on a panel of 24 asthma and COPD patients (9 in New York and 15 in Seattle) focused on the health effects of heart rate and pulmonary function associated with outdoor, indoor, and personal thoracic PM_10_ and PM_2.5_ exposure. In that study, forced expiratory volume in 1 sec (FEV1) and peak expiratory flow rate (PEF) via spirometry were measured, and symptom data were collected during a 12-day period both in the winter (New York and Seattle) and in the summer (New York) [22]. No consistent associations between particle pollution and lung function were found. De Hartog et al. conducted a multi-center panel study in 135 patients with asthma or COPD from Amsterdam, Athens, Birmingham, and Helsinki [21]. In that study, FVC, FEV1, and PEF were measured three times a day for a period of one week. Lung function was not consistently associated with PM_2.5_ or other fractions of particle pollutants, as measured at a central site in each of the above cities and both inside and outside the subjects’ residences. In Mexico City, a 10 μg/m^3^ increase in personal PM_2.5_ was associated with reductions in PEF and increased respiratory symptoms with a 2-day lag [23]. That study involved 29 COPD patients who were monitored for twelve days, three times over one year. In another, extensive study conducted in London, a panel of 115 COPD patients was monitored for an average period of 128 days. Daily information concerning respiratory symptoms and PEF measured with a peak flow meter were self-recorded. Gaseous and particle pollutants’ concentrations were measured through a personal monitor, specifically designed for that project. No association was observed between particulate matter and any outcome regarding respiratory performance. Paterson et al. [25] reviewed twelve studies investigating the effects of indoor PM_2.5_ and VOC exposure on asthma in adult patients. That review found insufficient evidence to connect PM_2.5_ and asthma in the indoor residence environment; however, there was sufficient evidence to associate VOCs, a primary pollutant that contributes to PM_2.5_, with increased asthma symptoms. The reasons for the differences observed among the various epidemiological studies are not certain. However, they could be associated with study design, e.g., insufficient monitoring periods or errors in exposure measurements, or with individual participants’ characteristics, such as time spent indoors, use of chemicals and cleaning products, activities resulting in PM emissions, etc.

The present study had to be conducted under certain constraints. First of all, it was confined to a small sample size, which may have limited its ability to establish associations between exposure to fine particles and respiratory health effects. Furthermore, the available sample size did not allow for an adjustment for the statistical analysis of confounders, such as temperature, gender, etc. Within that context, its results should be interpreted with caution. In addition, the present study monitored a single pollutant exposure, PM_2.5_, thus potentially not accounting for the impact of other gaseous or particulate pollutants on the lung function of asthmatic and COPD patients. Nevertheless, the selected pollutant has been acknowledged as one with the most adverse effects on health, with no established safety limit in terms of breathing.

On the other hand, the present study possessed a number of important advantages. Unlike other panel studies, measurements of pulmonary health and particle pollution parameters span over an entire year. This facilitated the assessment of the impact of fine particles on lung function over all seasons, in contrast to just one or two seasons, which was the case in most of the other published research. Moreover, the ability to assess lagged exposures facilitated the capture of effect estimates. Measuring PM_2.5_ concentrations inside and in the immediate outdoor environment of the subjects’ residences allowed for a more adequate representation and more accurate estimation of the actual levels of personal exposure to PM_2.5_, especially considering the substantial amount of time people spend indoors. The fact that pollution data in the present study were obtained by objective measurements, and not through estimations by modeling, minimized any possible exposure errors. Despite the detailed understanding of residential particle exposure that this study provides, future research employing greater sample sizes of susceptible sub-groups, or indeed of the general population, should be performed in order to assess health effects in residential environments.

## 5. Conclusions

The present study showed that the indoor concentration of fine particles exhibits high variability, depending on the various activities that take place inside a residence and the different behavioral patterns of the people who live in it. Results of this study revealed an increased exposure to PM_2.5_ both outdoors, in the ambience of a mid-sized city, but particularly in the indoor environment of a residence. The presence of smokers and the use of fireplaces seem to have an overwhelming impact on indoor air quality, whereas the duration of cooking activities seems to have an additional effect on the residential micro-environment. In particular: (a) ambient PM_2.5_ concentration levels were 7 times above WHO 2021 revised guidelines and 13 times above WHO guidelines inside the home environment; (b) PM_2.5_ exposure is associated with the reduction in PEF and occurrence of wheezing, in the indoor environment, with a significant effect especially during autumn; and (c) the importance of air-quality monitoring is highlighted when investigating the exposure to fine particle pollution and short-term health effects on vulnerable clusters of the population, as patients with chronic lung diseases.

This research provided a quantitative contribution to the existing findings on the relationship between respiratory health outcomes and fine particle exposure in a mid-sized city in Southeastern Europe, a geographical area that has received minimal research attention. By collecting an extended amount of respiratory health indicators per patient, such as PEF and symptoms, it enhanced the understanding of PM_2.5_ health effects while patients are still at home, in contrast to most other published work, which focuses on hospital admissions or emergency department visits.

It is essential to simplify the complex epidemiological findings and effectively disseminate their results to key stakeholders and decision makers, whereas urban planning should incorporate health issues in the local agenda, for instance in the infrastructure sector through controlling traffic load as well as by building key-partnerships between planning, public health, and other relevant teams. As regards the domestic environment, patients with chronic lung diseases should be informed by the treating clinician about the aggravating impact of indoor particulate matter and propose counter-active measures such as the reduction in ETS and use of open fireplaces, high-efficiency exhaust fans and chimneys in the kitchen, and use of air purifiers in order to reduce exposure to fine particles generated by their habits and behavioral patterns. As buildings become increasingly airtight for energy efficiency reasons, thus reducing the infiltration of airborne particles, their inhabitants will become increasingly susceptible to indoor-generated particle pollution. Therefore, it is essential to improve public awareness about the impact of various human activities on indoor air quality, as well as the importance of effective ventilation, in an effort to achieve lower personal exposure to air pollutants inside buildings and thus to improve the health of their more vulnerable inhabitants, since evidence suggests that they are intertwined.

## Figures and Tables

**Table 1:** Summary details of participants and sampling sites (Res_1 to Res_5 are the houses used as sampling sites).

Sampling Site	Asthma/COPD	Disease Severity	No of Residents	Floor/ Surface Area (m^2^)	Sampling Room Surface Area (m^2^)	Pets	Smoking Status	Household Activities with PM_2.5_ Generation	Cooker Type and Cooking Duration	Heating/Cooling System
Res_1	Asthma	GINA step 5	2	3rd 52	35	No	Never smoker	Cooking, candles, incense	Electric cooker <30 min	Natural gas/ air conditioning and Fan
Res_2	Asthma	GINA step 5	3	4th 110	60	No	Current smoker	cooking, smoking, fireplace use	Electric cooker 30–60 min	Oil central heating and fireplace/ air conditioning
Res_3	COPD	GOLD 2	3	1st 100	50	Yes	Current smoker	Cooking, smoking	Electric cooker 60–120 min	Oil central heating/ air conditioning
Res_4	Asthma	GINA step 5	2	Ground floor 65	20	No	Current smoker	Cooking, smoking	Electric cooker 60–120 min	Natural gas/ air conditioning and fan
Res_5	Asthma	GINA step 5	4	7th 130	40	Yes	Never smoker	Cooking, vaping	Electric cooker <120 min	Natural gas/ air conditioning

**Table 2:** Summary of fine particles concentration results (mean daily concentrations, calculated from 3 min timestamp values) and meteorological parameters (temperature, relative humidity).

	Overall	Winter	Spring	Summer	Autumn
Outdoor, n (%)	1337 (73.3%)	385 (85.6%)	380 (82.6%)	259 (56.3%)	313 (68.8%)
PM_2.5_ (μg/m^3^)					
Mean (±SD)	33.88 (±24.03)	51.52 (±29.59)	26.86 (±14.80)	18.77 (±6.61)	33.19 (±21.43)
Median (IQR)	27.14 (26.64)	44.42 (32.02)	23.96 (19.37)	18.22 (8.98)	28.89 (33.45)
Temperature (°C)					
Mean (±SD)	18 (±6)	12 (±2)	17 (±5)	29 (±3)	20 (4)
Median (IQR)	17 (12)	12 (3)	17 (8)	29 (4)	20 (7)
Relative Humidity (%)					
Mean (±SD)	45 (11)	49 (11)	44 (8)	39 (±9)	48 (1 ± 2)
Median (IQR)	45 (15)	48 (16)	44 (12)	38 (15)	48 (18)
Indoor, n (%)	1457 (79.8%)	397 (88.2%)	449 (97.6%)	303 (65.9%)	308 (67.7%)
PM_2.5_ (μg/m^3^)					
Mean (±SD)	63.89 (±68.41)	91.63 (±81.19)	64.11 (±72.31)	31.71 (±23.89)	59.50 (±58.86)
Median (IQR)	40.09 (54.61)	63.73 (80.96)	36.59 (50.20)	25.15 (26.57)	41.67 (52.29)
Temperature (°C)					
Mean (±SD)	24 (±3)	22 (±2)	23 (±2)	29 (±2)	24 (±3)
Median (IQR)	24 (6)	22 (3)	23 (4)	29 (3)	23 (6)
Relative Humidity (%)					
Mean (±SD)	46 (±8)	44 (±7)	46 (±7)	44 (±7)	53 (±10)
Median (IQR)	46 (12)	44 (10)	46 (10)	42 (9)	53 (17)
I/O ratio					
Mean (±SD)	2.20 (±2.10)	2.00 (±1.92)	2.82 (±2.73)	1.70 (±1.03)	1.94 (±1.62)
Median (IQR)	1.47 (1.81)	1.22 (1.95)	1.80 (2.40)	1.41 (1.46)	1.40 (1.53)
Hours indoors (%)	14.1 (58%)	17.8 (75%)	13.3 (54%)	11.6 (48%)	13.9 (58%)

**Table 3:** Yearly and season specific estimates for the association of PEF and fine particulate matter (PM_2.5_) at indoor and outdoor environment. Estimated change in PEF using linear mixed effect models. Decrease in PEF per unit increase in PM_2.5_ concentrations for same day up to 3 previous days.

	Yearly	Winter	Spring	Summer	Autumn
	Estimated change (95% CI) *p*-value	Estimated change (95% CI) *p*-value	Estimated change (95% CI) *p*-value	Estimated change (95% CI) *p*-value	Estimated change (95% CI) *p*-value
Home Outdoor					
lag 0	−5.133 (−10.222, −0.043) 0.048 *	−7.508 (−17.556, 2.539) 0.142	−1.264 (−10.285, 0.757) 0.782	24.906 (3.751, 6.061) 0.021 *	−7.531 (−18.078, 3.016) 0.160
lag 1	−6.407 (−11.369,−1.445) 0.011 *	−6.000 (−15.239, 3.238) 0.201	0.791 (−8.024, 9.607) 0.859	17.341 (−3.503, 38.186) 0.102	−14.342 (−24.809, −3.875) 0.007 *
lag 2	−5.874 (−10.913, −0.836) 0.022 *	−7.367 (−16.578, 1.845) (0.116)	2.723 (−6.041, 11.488) (0.540)	23.329 (4.467, 42.191) 0.016 *	−11.923 (−23.827, −0.019) 0.050 *
lag 3	−6.182 (−11.140, −1.224) 0.015 *	−7.487 (−16.761, 1.787) 0.113	1.636 (−7.031, 10.304) 0.710	21.338 (1.392, 41.284) 0.036 *	−11.718 (−22.408, −1.028) 0.032 *
Home Indoor					
lag 0	−1.902 (−6.625, 2.821) 0.429	2.146 (−10.116, 14.407) 0.731	−5.743 (−12.963, 1.476) 0.119	−4.891 (−17.542, 7.760) 0.447	−18.812 (−29.907, −7.717) <0.001 **
lag 1	7.975 (2.571, 13.379) 0.004 *	2.781 (−9.356, 14.919) 0.652	0.974 (−5.906, 7.854) 0.781	−6.793 (−19.677, 6.091) 0.300	−25.151 (−36.029, −14.274) <0.001 **
lag 2	9.960 (4.647, 15.274) <0.001 **	−1.585 (−13.848, 10.679) 0.799	1.309 (−5.631, 8.249) 0.711	−3.092 (−16.160, 9.976) 0.641	−17.615 (−28.510, −6.720) 0.002 *
lag 3	10.584 (5.165, 16.003) <0.001 **	−3.905 (−15.990, 8.180) 0.525	1.168 (−5.838, 8.174) 0.743	−0.128 (−13.593, 13.337) 0.985	−13.566 (−25.276, −1.855) 0.023 *

* *p* < 0.05, ** *p* < 0.001.

**Table 4:** Odds ratios (OR) with 95% confidence intervals for the biweekly occurrence of symptoms associated with a unit increase in ambient and indoor particulate matter with an aerodynamic diameter < 2.5 μm (PM_2.5_). OR were calculated by using generalized estimating equations (GEEs).

Symptoms	Odds Ratio	95% CI	*p*-Value
Indoor			
Cough	0.996	(0.992, 1.001)	0.115
Breathlesness	0.963	(0.941, 0.986)	0.002 *
Wheezing	1.004	(1.000, 1.009)	0.048 *
Ambient			
Cough	0.990	(0.966, 1.014)	0.402
Breathlessness	0.968	(0.935, 1.002)	0.063
Wheezing	1.004	(0.996, 1.012)	0.325

* *p* < 0.05.

**Table 5:** Yearly estimates for the association of AQLQ and ACT scores and fine particulate matter (PM_2.5_) at indoor and outdoor environment.

Questionnaires	Estimated Change	95% CI	*p*-Value
Indoor			
AQLQ	0.038	(−0.064, 0.140)	0.456
ACT	0.003	(−0.007, 0.013)	0.564
Ambient			
AQLQ	0.148	(−0.144, 0.440)	0.313
ACT	0.016	(−0.012, 0.044)	0.259

Abbreviations: AQLQ, Asthma-specific Quality of Life Questionnaire; ACT, Asthma Control Test. NOTE: AQLQ and ACT questionnaires include information from the past 2 weeks.

## Data Availability

The datasets used and/or analyzed during the current study are available from the corresponding author on reasonable request.
